# Identification of a second GTP-bound magnesium ion in archaeal initiation factor 2

**DOI:** 10.1093/nar/gkv053

**Published:** 2015-02-17

**Authors:** Etienne Dubiez, Alexey Aleksandrov, Christine Lazennec-Schurdevin, Yves Mechulam, Emmanuelle Schmitt

**Affiliations:** Laboratoire de Biochimie, Unité Mixte de Recherche 7654, Ecole Polytechnique, Centre National de la Recherche Scientifique, F-91128 Palaiseau cedex, France

## Abstract

Eukaryotic and archaeal translation initiation processes involve a heterotrimeric GTPase e/aIF2 crucial for accuracy of start codon selection. In eukaryotes, the GTPase activity of eIF2 is assisted by a GTPase-activating protein (GAP), eIF5. In archaea, orthologs of eIF5 are not found and aIF2 GTPase activity is thought to be non-assisted. However, no *in vitro* GTPase activity of the archaeal factor has been reported to date. Here, we show that aIF2 significantly hydrolyses GTP *in vitro*. Within aIF2γ, H97, corresponding to the catalytic histidine found in other translational GTPases, and D19, from the GKT loop, both participate in this activity. Several high-resolution crystal structures were determined to get insight into GTP hydrolysis by aIF2γ. In particular, a crystal structure of the H97A mutant was obtained in the presence of non-hydrolyzed GTP. This structure reveals the presence of a second magnesium ion bound to GTP and D19. Quantum chemical/molecular mechanical simulations support the idea that the second magnesium ion may assist GTP hydrolysis by helping to neutralize the developing negative charge in the transition state. These results are discussed in light of the absence of an identified GAP in archaea to assist GTP hydrolysis on aIF2.

## INTRODUCTION

In eukaryotic and archaeal cells, the initiator tRNA carrier is the e/aIF2 heterotrimer. In its GTP-bound form, this factor specifically binds Met-tRNA_i_^Met^ and brings it to the initiation complex. After start codon recognition, the factor, in its Guanosine diphosphate (GDP)-bound form, loses affinity for Met-tRNA_i_^Met^ and eventually dissociates from the initiation complex. This leaves Met-tRNA_i_^Met^ in the P site of the small ribosomal subunit and allows the final steps of the initiation process to occur. Hence, selection of the start codon is achieved through control of the nucleotide state of the factor (1,2).

e/aIF2 is composed of three subunits, α, β and γ. The γ subunit forms the core of the heterotrimer and contains the GTP-binding pocket. α and β are bound to γ but do not interact together. The γ subunit closely resembles elongation factor EF1A (3,4). As in the case of EF1A, productive binding of tRNA is GTP-dependent and related to the ON conformations of two regions of the G domain called switch 1 and switch 2 (5). Despite this strong homology, recent studies have shown that the tRNA binding modes strongly differ in EF1A and e/aIF2 (6).

In eukaryotes, the nucleotide state of eIF2 is regulated with the help of several initiation factors. A 43S pre-initiation complex, comprising at least the small ribosomal subunit, the initiator tRNA and the eIF1, eIF1A, eIF2, eIF3 and eIF5 factors, binds the mRNA at its 5′ capped-end to form a 48S complex (7–9). The pre-initiation complex then scans the mRNA until an AUG start codon is found. eIF1 and eIF1A are important to promote an open, scanning-competent, 48S complex (10–13). By facilitating GTP hydrolysis on eIF2, the eIF5 factor plays the role of a GTPase-activating protein (GAP; 14–16). Release of the inorganic phosphate produced by GTP hydrolysis, rather than hydrolysis itself, appears to be the key event triggered by recognition of a start codon (2,17). In this view, the eIF1 factor would prevent translational starts on non-AUG codons by blocking Pi release from eIF2. In turn, upon start codon recognition, conformational changes in the pre-initiation complex would promote the release of eIF1 and that of the Pi group (2,10,13,17). After release of eIF2:GDP, a guanine nucleotide exchange factor, eIF2B, is required for the recycling of eIF2:GDP into eIF2:GTP.

In archaea, scanning does not occur and the initiation codons are located either in the vicinity of a Shine–Dalgarno sequence, or near the 5′ end of mRNA (18). Orthologs of eIF1, eIF1A and eIF2 exist (19). This suggests that the molecular events that accompany start codon recognition are very similar in both archaea and eukaryotes. On the other hand, eIF3, eIF5 and eIF2B have no orthologs in archaea. Thus, GTP hydrolysis on archaeal aIF2 is thought to occur without GAP assistance and the recycling of aIF2:GDP into aIF2:GTP is thought to be spontaneous. However, to date, no GTPase activity for archaeal IF2 was reported (20).

The γ subunit of e/aIF2 contains the guanine nucleotide-binding pocket delineated by regions specifically encountered in all G-proteins (GKT loop, SW1 and 2, QNKIE and SALH sequences). Sequence alignments with other GTPase translation factors indeed show a high level of sequence conservation within these regions. In particular, the catalytic histidine residue in the switch 2 region crucial for GTP hydrolysis is strictly conserved in all GTPase translation factors (3).

Important steps forward in understanding GTP hydrolysis by translational GTPases were obtained thanks to the high-resolution crystallographic structures of the ribosome-bound states of EF-Tu and EF-G (21–23), although mechanistic details cause controversy (24–26).

e/aIF2 is peculiar in that it functions on the small ribosomal subunit, whereas other translational GTPases bind the same region of the assembled ribosome in all species and likely use the sarcin-ricin loop in the large subunit for activation of GTP hydrolysis. In the present study, we were interested in GTP hydrolysis by archaeal aIF2 from *Sulfolobus solfataricus* (Ss-aIF2). We were able to observe GTP hydrolysis by aIF2 or by its isolated γ subunit. Mutation of the catalytic H97 residue into alanine reduced the rate of hydrolysis by one order of magnitude. High-resolution crystal structure of the H97A mutant in the presence of GTP revealed the presence of a second magnesium ion bound to GTP. Binding of this second magnesium ion involves the highly conserved D19 residue from the GKT loop and the main chain atoms of G44 from SW1. Accordingly, mutation of D19 into alanine also reduced the rate of GTP hydrolysis by one order of magnitude. Finally, the role of the second magnesium ion was investigated using molecular simulation techniques. Free energy simulations suggest that in the presence of the second magnesium ion the catalytic histidine prefers to be neutral. The hybrid potential free energy simulations show that the presence of the second magnesium ion strongly activates GTP hydrolysis in aIF2. These results are discussed in light of the absence of an identified GAP in archaea to assist GTP hydrolysis on aIF2.

## MATERIALS AND METHODS

### GTPase assays

D19A, H97A and D19AH97A mutants of *S. solfataricus* aIF2γ were obtained via polymerase chain reaction mutagenesis of plasmid pET15b-Ss-aIF2γ according to the QuikChange^TM^ site-directed mutagenesis method (Stratagene). Wild-type and variants of aIF2 and aIF2γ subunits were purified as described (27). Methionyl-tRNA_f_^met^ (A1-U72) was prepared as described (28). GTPase activities were assayed as follows: aIF2 and Met-tRNA_f_^Met^(A1-U72) were mixed (400 nM each, final concentrations) in a buffer containing 10 mM HEPES pH7.5, 200 mM NaCl, 5 mM MgCl_2_, 10 mM 2-mercaptoethanol and pre-incubated 2 min at 65°C. Reaction was started by adding γ[^32^P]-GTP to a final concentration of 768 pM and further incubated at 65°C. From zero time, aliquots of this mixture were periodically mixed with 1 ml of 0.35% perchloric acid in 50 mM sodium acetate pH 4.5 containing 0.4% w/v activated charcoal plus 100 mM PPi. After adsorption of GTP, the mix was filtered on Whatman paper and the retained radioactivity was counted on a Beckman LS 6500 scintillation counter. The same procedure was used with the aIF2γ subunit alone or with the variants of aIF2γ. When stated, aminoacyl-tRNA was omitted and replaced by reaction buffer. The GTP hydrolysis curves as a function of time were fitted with a single exponential, $[{\rm GTP}{\rm }] = [{\rm GTP}{\rm }]_0 e^{ - k_{{\rm obs}{\rm }} t}$, using the MC-Fit program (29). Reported results are mean ± s.d. from at least three independent experiments. Spontaneous GTP hydrolysis rate in the absence of aIF2 in the assay was lower than 0.003 min^−1^. With the three studied enzymes (WT, H97A and D19A), in the presence and in the absence of tRNA, we have verified that increasing four times the concentrations of aIF2 (and tRNA when applicable) or of aIF2, tRNA (when applicable) and γ[^32^P]-GTP did not result in a change in the observed rate constants, indicating that GTP hydrolysis is first order under these conditions and not rate limited by a binding event (2). No γ[^32^P]-GTP hydrolysis was detected during the first few seconds of the reaction, thereby excluding the occurrence of an initial burst.

### GDP binding assay

The nucleotide-binding assay was performed essentially as described (20). The purified aIF2 proteins (30 picomoles) were incubated 10 min at 65°C in a buffer containing 10 mM HEPES pH7.5, 200 mM NaCl, 5 mM MgCl_2_, 10 mM 2-mercaptoethanol with increasing amounts (0–464 picomoles) of [^3^H]GDP (∼18 000 d.p.m/pmol). The final volume of the reaction was 50 μl. Aliquots (20 μl) were withdrawn and mixed with 1 ml of cold incubation buffer and then filtered through Millipore 0.22 μM nitrocellulose disks, which were washed with 2 ml of cold incubation buffer. The filters were then counted by liquid scintillation in a Beckman LS 6500 counter. Results were fitted with simple binding curves from which apparent dissociation constants and their associated standard errors were derived using the MC-Fit program (29).

### Crystal structures

After purification, aIF2γ solutions were stored in a buffer containing 10 mM HEPES pH 7.5, 200 mM NaCl, 10 mM 2-mercaptoethanol. Before crystallization trials, nucleotides were added to the protein at a final concentration of 10 mM in the presence of 10 mM MgCl_2_. Depending on the trial, either GTP, GDP or GDPNP was used. Highly diffracting crystals of aIF2γ were obtained using 50–70% methyl pentanediol (MPD) as a precipitating agent in the presence of 10 mM HEPES pH 7.5. Before data collection, crystals were flash-cooled in liquid nitrogen. Diffraction data were collected at 100 K (λ = 0.984 Å) on the Proxima-1 beamline at the SOLEIL synchrotron (Saint-Aubin, France) equipped with a Pilatus detector. Diffraction images were analyzed with X-ray detector software (XDS) (30), and the data were processed with programs of the CCP4 package (31). The structure of wt-aIF2γ bound to GDPNP and that of wt-aIF2γ bound to GDP (arising from GTP hydrolysis) were solved by molecular replacement with PHASER (32) and the previously determined structure of aIF2γ (PDB ID, 2AHO, (27)). Coordinates and associated B factors were refined through several cycles of manual adjustments with COOT (33) and positional refinement with PHENIX (34). According to crystal isomorphism, the resulting structure of wt-aIF2γ bound to GDPNP was used as a starting model (after removal of GDPNP and water molecules) to refine the models corresponding to D19A and H97A mutants. The following refinement procedures were the same as the one described for wt-aIF2γ. B factors for protein atoms were refined anisotropically and those for waters were refined isotropically, except for the wt-aIF2γ-GDP dataset for which all B factors were refined isotropically. Final statistics are shown in Table 2.

**Table 1.** Rate constant for GTP hydrolysis

	*k*_obs_ (min^−1^) − Met-tRNA	*k*_obs_ (min^−1^) + Met-tRNA
γ	0.35 ± 0.02	0.38 ± 0.02
αγ	0.40 ± 0.01	0.45 ± 0.01
βγ	0.42 ± 0.03	0.49 ± 0.03
αβγ	0.47 ± 0.01	0.58 ± 0.02
γH97A	0.034 ± 0.008	n.d
αβγH97A	n.d	0.023 ± 0.008
γD19A	0.031 ± 0.008	n.d
αβγD19A	n.d	0.043 ± 0.005
γH97AD19A	<0.007	<0.007

n.d: not determined.

The GTP hydrolysis curve as a function of time was fitted with a single exponential using the MC-Fit program (29). Each experiment was independently repeated at least three times. Reported results are mean ± s.d. from at least three independent experiments.

**Table 2.** Data collection and refinement statistics

Data collection	wt-aIF2γ−GDPNP	wt-aIF2γ−GDP	D19A-GTP_4_	D19A-GDPNP_4_	D19A-GTP_24_	H97A-GTP_4_	H97A-GDPNP_4_	H97A-GTP_24_
Space group	P 2_1_2_1_2_1_	P 2_1_2_1_2	P 2_1_2_1_2_1_	P 2_1_2_1_2_1_	P 2_1_2_1_2_1_	P 2_1_2_1_2_1_	P 2_1_2_1_2_1_	P 2_1_2_1_2_1_
Cell dimensions								
a, b, c (Å)	46.4 60.9 143.4	75.9 120.0 53.4	46.5 60.25 144.4	46.4 60.9 143.7	45.9 59.9 144.2	46.3 60.6 144.6	46.3 60.5 144.3	46.3 60.5 144.5
α,β,γ(°)	90.0 90.0 90.0	90.0 90.0 90.0	90.0 90.0 90.0	90.0 90.0 90.0	90.0 90.0 90.0	90.0 90.0 90.0	90.0 90.0 90.0	90.0 90.0 90.0
Resolution (Å)	46.44–1.3	48.82–1.94	46.25- 1.65	46.46–1.43	46.06–1.71	46.4 -1.50	46.34–1.58	46.36–1.69
*R*_sym_	5.4 (54.9)	9.1 (75.5)	7.9 (72.8)	12.3 (75.2)	14.4 (61.7)	13.6 (53.3)	8 (81.6)	7 (56)
I/σI	12.18 (1.74)	11.95 (1.95)	15.39 (2.64)	9.43 (2.12)	8.31 (2.46)	8.66 (2.25)	13.3 (1.97)	12.85 (1.74)
Completeness(%)	97.5 (90.9)	98.7 (96.3)	99.5 (97.7)	99.8 (99)	99.4 (96.9)	99.7 (98.4)	99.4 (97.2)	97.3 (91.9)
Redundancy	3.45 (3.28)	4.39 (4.44)	7.12 (6.87)	7.17 (6.86)	6.19 (6.08)	6.42 (7.15)	7.16 (7.08)	3.44 (2.7)
**Refinement**								
No. Reflections	98005	36252	49650	76298	43358	65988	55644	45471
*R*_work_/*R*_free_	0.1669/0.1972	0.1741/0.2289	0.1482/0.1874	0.1513/0.1888	0.1455/0.203	0.1591/0.1888	0.145/0.2022	0.1605/0.223
No. atoms								
Protein	3334	3235	3328	3282	3262	3294	3285	3216
Ligand	64	27	68	64	56	60	64	61
Waters	419	339	358	408	349	317	381	310
B factors (Å^2^)								
Protein	23.6	28.8	27.1	27.0	31.0	31.5	29.2	31.4
Ligand	GDPNP_1_ 16.9 Mg_1_ 16.1 GDPNP_2_ 59.6	GDP 18.9 Mg_1_ 17.3	GTP 25.5 Mg_1_ 21.2 GDP 90.0	GDPNP_1_ 20.9 Mg_1_ 17.35 GDPNP_2_ 84.8	GDP_1_ 24.7 Cl^−^ 24.5 Mg_1_ 24.2 GDP_2_ 84.8	GTP_1_ 29.7 Mg_1_ 23.1 Mg_2_ 46.2 GDP_2_ 66.5	GDPNP_1_ 26.1 Mg_1_ 18.4 GDPNP_2_ 90.5	GDP_1_ 29.7 PO_4_ 31.1 Mg_1_ 25.2 GDP_2_ 86.0
Waters	35.2	36.7	37.7	39.8	40.7	41.0	41.2	40.3
Bond length (Å)	0.006	0.008	0.007	0.006	0.007	0.006	0.006	0.007
Bond angles (°)	1.135	1.120	1.086	1.100	1.082	1.107	1.039	1.074

Values in parentheses are for the highest resolution shell. Note that for D19AGDPNP, D19AGDP and H97AGTP datasets the relatively high global *R*_sym_ values were due to the combination of very low crystal mosaicities (ca. 0.07°) and to slight beam instability on the day of data collection. This has no detectable consequence on the quality of the refinement.

Ligands are indicated. Subscript 1 or 2 indicates ligand at the catalytic binding site on γ domain I or at the weak binding site on γ domain II.

### Molecular dynamics simulations

The simulations included protein residues within a 24 Å sphere, centered on the nucleotide-binding site. With this radius of the sphere, good results were produced in previous studies of the GTP hydrolysis in the EF-Tu:ribosome system (24). Protonation states of histidines were assigned by visual inspection, except H97, which protonation state was specifically studied in this work. Protein atoms between 20 and 24 Å from the sphere's center were harmonically restrained to their experimentally determined positions. The histidine residue was positioned in place of the alanine at position 97. This was done by superimposing the GTP ligands of the aIF2 and EF-Tu:ribosome systems (PDB entries 2XQD and 2XQE (21)) and extracting the appropriate histidine residue, H85.

In addition to crystal waters, a 75-Å cubic box of water was overlaid, and waters overlapping the protein were removed. Periodic boundary conditions were assumed; i.e. the entire 75-Å box was replicated periodically in all directions. All long range electrostatic interactions were computed efficiently by the particle mesh Ewald method (35), and the appropriate number of potassium counterions was included to render the system electrically neutral. Molecular dynamics (MD) simulations were performed at constant room temperature and pressure, after 200 ps of thermalization. The CHARMM27 force field was used for the protein (36,37) and the TIP3P model for water (38). Calculations were done with the NAMD program (39). Ten nanosecond of unrestrained MD was performed at constant room temperature and pressure.

To create a starting structure of the aIF2γ:GTP complex with one magnesium ion, we manually eliminated from the crystal structure the second magnesium ion, which does not interact directly with γ- and β-phosphates of GTP.

To compute the protonation free energy of the GTP gamma phosphate and H97, we used a Poisson–Boltzmann Linear Response Approximation or PB/LRA (40). The setup of simulations was similar to that used in previous studies (40,41). Details of the method are given in Supplementary data.

### Hybrid potential simulations

For the hybrid potential QM/MM calculations, the aIF2:GTP complex was partitioned between QM and MM regions. The QM region had 59 atoms, comprising the GTP, two Mg cations, the side chain of H97, the hydrolytic water molecule, the side chain of D19, two water molecules which interact with the Mg ion of GTP and four water molecules that interact with the second Mg ion. The MM region had around 40 000 atoms. The QM region was electrically neutral.

The atoms in the MM region were represented with the CHARMM27 force field (36,42), whereas a density functional theory (DFT) method was used for the QM region with the BLYP functional and Ahlrichs's split valence basis with polarization functions (43). Single point calculations on the optimized structures were done with the larger def2-TZVP basis set (44), which has polarization functions on all atoms, and MP2 method. All geometry optimizations and reaction path calculations were performed with the QM(DFT)/MM potential.

The starting coordinates of the complexes were taken after 1.0 ns of MD simulation on the systems described in section MD Simulations. The QM(DFT)/MM calculations were performed with the pDynamo software (45), and its interface to the ORCA program (46). No truncation or cutoff was employed to calculate non-bonding interactions. The reaction path was optimized with the Nudged Elastic Band (NEB) method (47) implemented in pDynamo. The utility of the NEB method is that it does not require a predefined set of reaction coordinate variables and makes no assumptions as to how the reaction proceeds. The only bias in the calculation comes from the structures employed in the starting guess for the NEB pathway. After a regular NEB calculation was converged, the climbing image method (CI-NEB) was applied to the image with the highest energy in the path. Previously we showed that application of the CI-NEB is required for fully refined saddle point structures (47).

Finally, to estimate free energies we used the free energy perturbation (FEP) method of Kästner *et al*. (48), which we used in a previous study (49). Details of the method and simulations are given in Supplementary data.

## RESULTS

### GTP hydrolysis by aIF2

Very little is known about how aIF2 hydrolyses GTP. As a first step in the study of the mechanism of GTP hydrolysis, we measured the rate constants for GTP hydrolysis without ribosomal subunits on the complete aIF2 heterotrimer or on aIF2 subcomplexes in the presence or in the absence of Met-initiator tRNA. As shown in Table 1, the rate constant for GTP hydrolysis by full aIF2 in the presence of Met-tRNA is 0.58 ± 0.02 min^−1^. Minor effects resulting from the presence of the Met-tRNA or of the α and β subunits bound to the core γ subunit were observed (Table 1, Supplementary Figure S1). Indeed, a similar rate value was measured with the isolated gamma subunit (0.38 ± 0.02 min^−1^). In order to ensure that the measured rates of hydrolysis were not limited by a binding event, we verified that increasing the concentrations of all the components at least 4-fold did not result in a change in the observed rates (see Materials and Methods). Interestingly, these catalytic rate values were close to that measured for the eukaryotic ternary complex in the presence of eIF5 (TC.eIF5; 0.42 ± 0.03 min^−1^), whereas the rate of hydrolysis was about 5x10^−4^ min^−1^ in the absence of eIF5 (2). This shows that aIF2 has indeed a higher intrinsic GTPase activity than its eukaryotic counterpart. Moreover, since similar rates were measured for the full heterotrimer, in the presence or absence of Met-tRNA, and for the isolated γ subunit, it is likely that features of the isolated γ subunit are mostly responsible for this intrinsic catalytic activity (Table 1, Supplementary Figure S1).

We obtained high-resolution diffracting crystals of the isolated γ subunit using MPD as a precipitating agent. Crystals were obtained in the presence of GDP-Mg^2+^ or in the presence of GDPNP-Mg^2+^ (Table 2). The structure of wt-aIF2γ-GDPNP-Mg^2+^ was refined to 1.3 Å resolution. All residues are visible and two nucleotides are bound to aIF2γ. One GDPNP-Mg^2+^ molecule is bound to the canonical binding pocket in the G-domain and a second GDPNP molecule is found in a pocket within aIF2γ domain II corresponding to the binding site of the terminal A76 base of the initiator tRNA ((6) Supplementary Figure S2). No magnesium ion is liganded to this second GDPNP molecule. Notably, this second GDPNP binding site was already observed in another Ss-aIF2-GDPNP structure (50).

**Figure 1.**
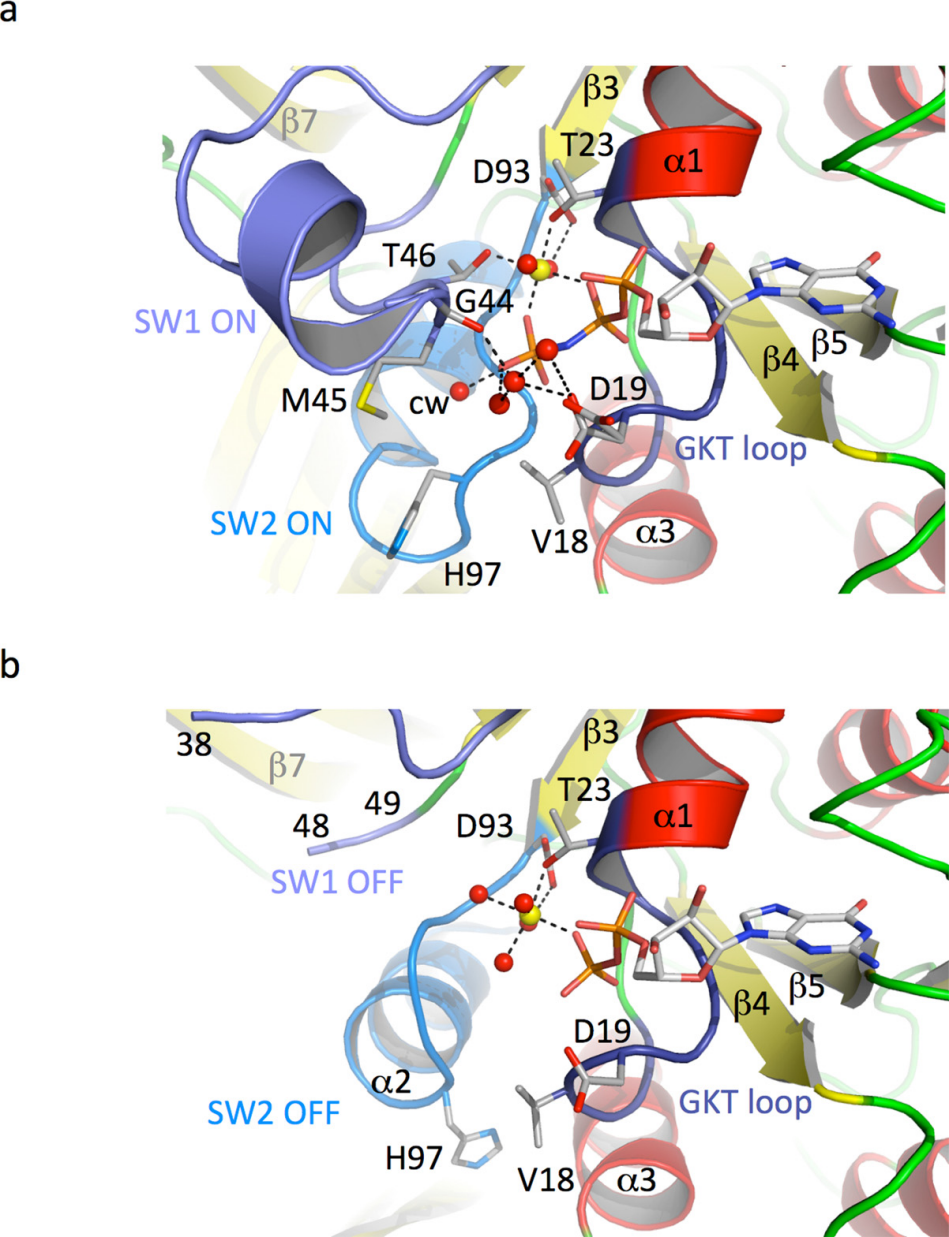
The nucleotide-binding pocket of Ss-aIF2γ. (**a**) Structure of wild-type Ss-aIF2γ bound to GDPNP-Mg^2+^. The protein is shown in cartoons. GKT loop is deep blue, SW1 is slate blue and SW2 is light blue. Residues surrounding phosphate groups of GDPNP are shown in sticks and labeled. The magnesium ion is shown with a yellow sphere and water molecules are shown with red spheres. The catalytic water is labeled cw. The relevant network of interactions is shown with dashed lines. (**b**) Structure of wild-type Ss-aIF2γ bound to GDP-Mg^2+^. The color code used is the same as in panel a. SW1 region is partly disordered from residues 39–47. The nomenclature of secondary structures is as in (27).

The canonical GDPNP-Mg^2+^ is bound by residues belonging to the conserved sequences forming the nucleotide-binding pocket of all G-binding proteins (Figure 1a). No packing interactions are involved in the binding of the nucleotide. Switch 1 and switch 2 regions are in the ON state. Interestingly, the switch 1 region is fully defined in the electron density map and its conformation is totally reminiscent of that observed for the switch 1 region in the active conformation of EF1A (Supplementary Figure S3 (5,51)). In particular, residues 39–44 adopt a helical conformation. Notably, such a conformation for the switch 1 region was already observed in the structure of the ternary initiation complex aIF2-GDPNP-Met-tRNA ((6) and Supplementary Figure S5). In wt-aIF2γ-GDPNP-Mg^2+^, the octahedral coordination of a tightly bound magnesium ion is clearly visible. In the equatorial plane, four bonds involve T23 from the GKT loop, T46 from the switch 1 region and two oxygens from the β and γ phosphate groups. Two water molecules are in apical positions. One of them is stabilized by D93 from switch 2 (Figure 1a).

Comparison of the structure with the wt-aIF2γ-GDP-Mg^2+^ structure shows the transition between switch ON and switch OFF states (Figure 1). In the GDP-bound structure, the magnesium ion is still bound but three water molecules and one oxygen of the β phosphate define the equatorial plane, whereas another water molecule and the hydroxyl group of T23 are in apical positions with respect to Mg^2+^ (Figure 1). Changes of the coordination of the magnesium ion are correlated to the structural change following the loss of the γ-phosphate previously termed the loaded-spring mechanism (52). Overall, the conformation of wt-aIF2γ−GDPNP differs from that of wt-aIF2γ-GDP by a rotation of domains II-III with respect to domain I by ca. 14°. This rotation is partly due to the movement of switch 2 helix, and to the subsequent remodeling of its interaction network with β17 and the following loop of domain III (Supplementary Figure S4). In addition, transition of switch 1 position from ON to OFF causes release of interactions between domains I and II involving the strictly conserved R219 residue (Supplementary Figure S5d and e). Interestingly, this residue also interacts with GDPNP bound to domain II, therefore contributing to the secondary binding site. In GDP-bound structures (present work and PDB ID 4M0L, (50)), the secondary site was not occupied. In these GDP-bound structures R219 does not contact domain I (Supplementary Figure S5d) and is not poised to interact with a secondary nucleotide. As the secondary nucleotide mimics the terminal adenosine of the initiator tRNA (6), this suggests that domain movements and positioning of R219 contribute to the release of aIF2 from the tRNA after Pi release (Supplementary Figures S2 and S5).

In wt-aIF2γ-GDPNP-Mg^2+^, the catalytic water is located 3.55 Å from the P atom of the γ phosphate. As in EF-Tu, the catalytic water is held in position by hydrogen bonding with main chain atoms corresponding to the carbonyl (CO) group of T46 (switch 1) and the amino (NH) groups of G96 and H97 (switch 2) (51). H97, equivalent to the catalytic H85 of EF-Tu from *Thermus thermophilus* (53), is oriented away from the water molecule in its non-activated conformation (Figure 1a). The positions of the side chains of M45 and V18 forming the so-called ‘hydrophobic gate’ might have impaired its motion toward the catalytic water (5,51). Moreover, hydrogen bonds of H97 Nδ with backbone NH groups of E98 and V99 stabilize the inactive conformation of H97. Two alternative conformations of D19 from the GKT loop are observed, showing that its position is not firmly stabilized. However, a water-mediated interaction connecting the γ-phosphate group of GDPNP to the side chain of D19 is observed for both conformations. Moreover, a tightly bound water molecule is located between the main chain carbonyl groups of G44 and D19.

### H97A and D19A variants

In order to evaluate their roles in GTP hydrolysis, H97A and D19A variants of aIF2γ were constructed. The double mutation was also introduced to obtain a D19AH97A variant. Both mutated aIF2γ subunits and full heterotrimeric aIF2 variants were purified. In order to verify that mutation of H97 or D19 did not change the affinity of the mutated protein for the nucleotide, GDP binding affinities were measured using a nitrocellulose filter-binding assay for wt-aIF2 and for the two variants H97A-aIF2 and D19A-aIF2. The apparent *K*_d_ values were of 0.52 ± 0.12 μM, 0.34 ±0.10 μM and 0.24 ± 0.10 μM for wt-aIF2, H97A-aIF2 and D19A-aIF2, respectively. These results showed that the mutations did not strongly modify the GDP binding properties of the two mutant enzymes. GTPase activities were measured for the three variants. Mutations D19A and H97A each lowered the rate of GTP hydrolysis by a factor of 8.5 and 17.5, respectively (Table 1). With D19AH97A variant the rate became too low to be measurable (Table 1) thereby suggesting that the two residues participate in an additive manner to GTP hydrolysis. As in the case of the wild-type factor, the rate values were not modified upon increasing the concentrations of all components by a factor of four, showing that these low rate values were not limited by a binding event.

**Figure 2.**
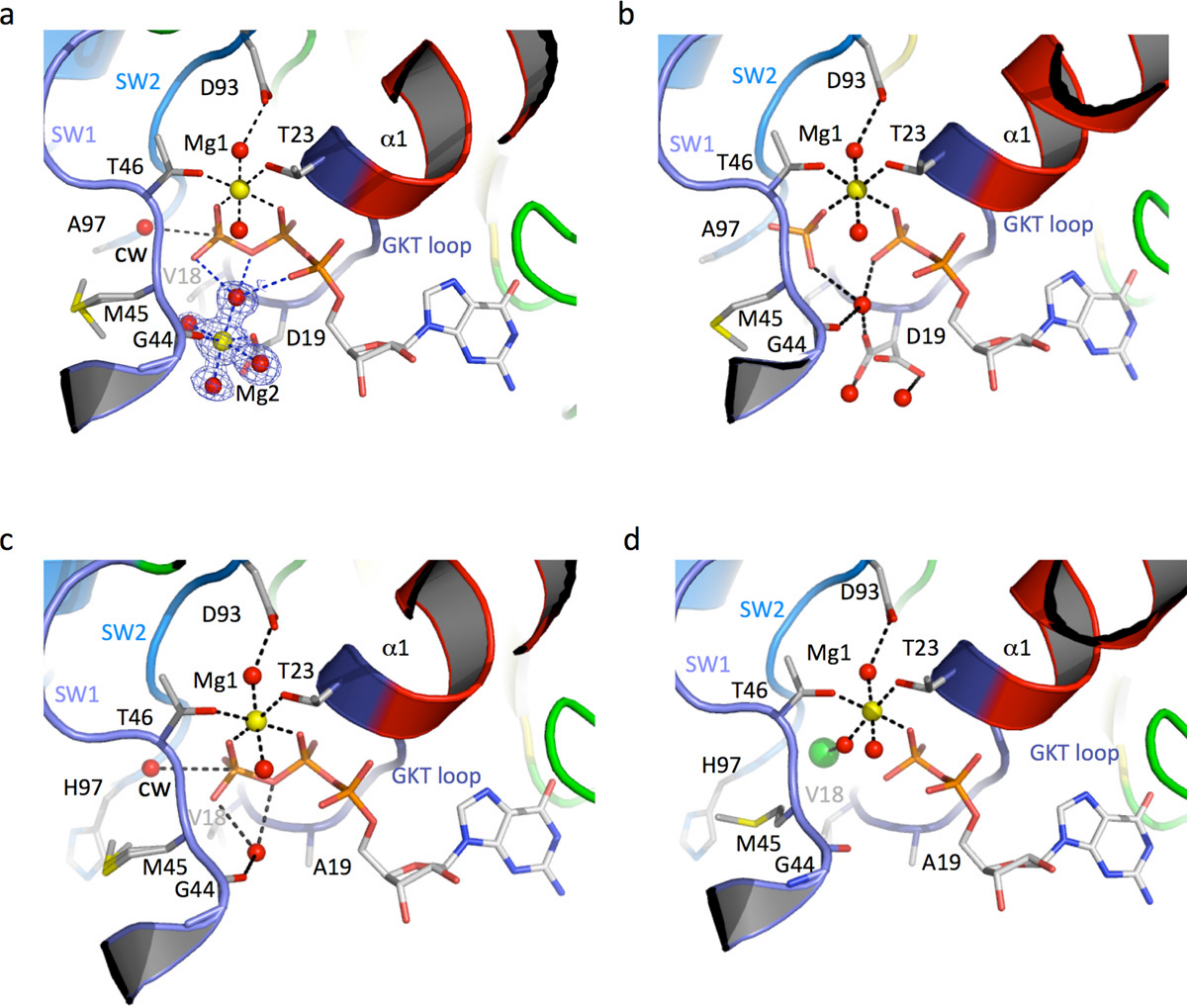
aIF2γ H97A and D19A crystal structures. The color code used is the same as in Figure 1. The views were drawn using a same orientation. (**a**) Structure of H97A-GTP_4_. The 1.5 Å resolution ‘2Fo-Fc’ map contoured at 1.0 standard deviation showing the coordination sphere of the second magnesium ion is drawn using the carve command of Pymol. (**b**) Structure of H97A-GTP_24_. (**c**) Structure of D19A-GTP_4_. (**d**) Structure of D19A-GTP_24_. A Cl^−^ ion is shown as a green sphere.

H97A and D19A mutants were crystallized in the same conditions as those used for wt-aIF2γ but in the presence of GTP-Mg^2+^. Highly diffracting crystals isomorphous to wt-aIF2γ−GDPNP crystals (Table 2) were obtained at 4°C and at 24°C. The corresponding structures will hereafter be named with a subscript, 4 or 24, corresponding to the temperature of crystal growth. In keeping with the observed decreased rates of GTP hydrolysis (Table 1), we observed that GTP was intact within the crystals grown at 4°C. In the D19A-GTP_4_ and H97A-GTP_4_ structures, GTP is tightly bound to the canonical nucleotide-binding pocket in a position similar to that of GDPNP in wt-aIF2γ (Figure 2a and c and Supplementary Figure S6). Unexpectedly in H97A-GTP_4_ structure, a second magnesium ion (hereafter named Mg_2_) is observed within the nucleotide-binding pocket. The octahedral coordination of the second magnesium ion is clearly visible (Figure 2a). Four water molecules form the equatorial plane. One of these water molecules also interacts with oxygen atoms of the α, β and γ phosphate groups of GTP (Figure 2a). In apical positions are the side chain of D19 and the carbonyl group of G44. No alternate conformation of D19 is observed showing that the residue is now tightly stabilized. When GDPNP is used in crystallization (H97A-GDPNP), no second magnesium ion is observed and alternative conformations of D19 are visible, like in the wild-type γ−GDPNP structure (Supplementary Figure S6b). This observation might be linked to the previous observations that for some translation GTPases, affinity of GDPNP is weaker than affinity of GTP and thereby that GDPNP is not a perfect mimic of GTP (54,55). If D19 is mutated to alanine (D19A-GTP_4_ structure), the second magnesium is not observed in keeping with the involvement of D19 in Mg_2_ coordination. One water molecule is still bound to G44 and to the β and γ phosphates of GTP. The catalytic H97 is in the non-activated conformation and GTP is intact (Figure 2c).

**Figure 3.**
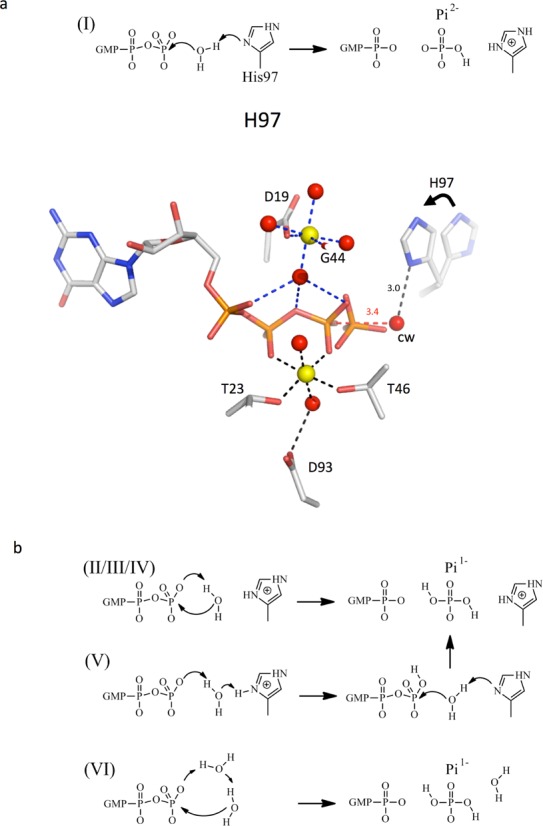
Schematics of the mechanisms investigated in the QM/MM-FEP simulations. (**a**) Scheme of mechanism I corresponding to that proposed by (21) and structural model. The structures corresponding to H97AGTP_4_ and _24_ were superimposed. GTP, Magnesium ions and their ligands are from H97AGTP_4_. The Pi group from H97AGTP_24_ is superimposed in order to highlight Walden inversion at the γ phosphoryl group upon GTP hydrolysis into GDP and Pi. H97 was modeled in its inactive position using superimposed wt-aIF2γ-GDPNP structure and in its active position using EF-Tu ribosome complex (PDB ID Code 2QXD, (21)). The catalytic water is shown as observed in H97AGTP_4_, at a distance of 3.4 Å from the γ phosphorus. Its distance to the Nδ of H97 modeled in the active position is 3.0 Å. (**b**) Other studied mechanims. Mechanism II was suggested by (25,58). Mechanisms III and IV (not shown) are variants of mechanism II in which H97 is in its neutral states protonated on Nδ and Nϵ respectively. Mechanism V is the mechanism proposed by (24).

In contrast, with both the H97A and D19A mutants, according to their weak residual GTPase activity, the conversion of GTP into GDP took place in the crystals at 24°C (Figure 2b and d, Table 2). In H97A-GTP_24_ structure, the Pi group remains tightly bound to aIF2γ and retains the classical coordination sphere of the GTP γ-phosphate as observed in the H97A-GTP_4_ structure. However, Mg_2_ is released, but a water molecule contributes to stabilize the γ-phosphate (Figure 2b). Superimposition of HA97_4_ and HA97_24_ structures visualizes the inverted configuration of the Pγ that is postulated from the S_N_2 mechanism (Figure 3 and Supplementary Figure S6c; (56)). The H97A-GTP_24_ structure also shows that hydrolysis can take place without H97 and reinforces the proposed role for D19 in catalysis. Accordingly, the high quality electron density maps suggested that in H97A-GTP_4_, a very small fraction of GTP was converted into GDP-Pi. Finally, the D19A-GTP_24_ structure shows that GTP is hydrolyzed. Pi group is no longer bound to aIF2γ but a Cl^−^ ion is bound at the corresponding position (Figure 2d). Possibly, removal of the D19 side chain has cleared the way to the surface of the protein and allowed rapid diffusion of the Pi group. In the H97A-GTP_24_ and D19A-GTP_24_ structures, no movement of the switch region is visible consistently with isomorphism of the crystals (Table 2). Hence, these structures suggest that GTP hydrolysis can occur without movement of the switch regions. Finally, movements of the side chain of M45 are visible upon comparison of all available structures (Figure 2). Such motions might accompany the diffusion of a water molecule required for activation of GTP hydrolysis.

Overall the crystallographic data as well as rate measurements suggest that D19 has an important role in GTP hydrolysis. Its role could be complementary to that of H97, as suggested by the additive effect of the mutations in the H97AD19A variant (Table 1). According to the presence of Mg_2_ bound to D19 it is tempting to propose that Mg_2_ would help to neutralize the developing negative charge on the transition state.

### H97 and GTP protonation free energy simulations

In order to further study a possible relevance of the second magnesium ion in GTP hydrolysis by aIF2γ, molecular simulation techniques were used. Several mechanisms where H97 and a catalytic water molecule have essential roles, by analogy to the case of EF-Tu, were tested. In the first step, free energies of histidine and GTP protonations in the presence of Mg_1_ or of Mg_1_ and Mg_2_ were computed as described (24) by PB/LRA method. Corresponding free energies are given in Table 3.

**Table 3.** PB/LRA free energy simulations comparing different protonation states of H97 and GTP

Protonation	Protonation	Mg ions	
GTP	H97	Mg_1_	Mg_1_+Mg_2_
−4	His*ϵ*	0.0	0.0
−4	(+)	0.5	3.0
−3	His*ϵ*	1.1	7.5
−3	(+)	4.1	13.9

Free energies are in kcal/mol.

The state with a neutral N*ϵ* H97 and GTP deprotonated on the γ phosphate was chosen as a reference state. The protonation free energy of H97 is 0.5 kcal/mol in the absence of Mg_2_. In the presence of Mg_2_ the protonation energy is 3.0 kcal/mol. In the latter case, the neutral N*ϵ* form of H97 is clearly favored. The positive environment due to Mg_2_ likely explains the destabilization of a positively charged form of H97.

With Mg_1_ only, the protonation free energy of GTP is 1.1 ± 0.2 kcal/mol in aIF2γ with a neutral H97, which slightly favors the state with a deprotonated GTP. In solvent, γ- phosphate pKa of NTP:Mg is 4.7 (57), which corresponds to the protonation free energy in the standard state of 3.1 kcal/mol. A decreased protonation free energy in the protein can be understood by electrostatic interaction between the γ phosphate of GTP and D19. In the presence of Mg_2_, the protonation free energy is strongly increased to 7.5 kcal/mol. Finally, the state with a biprotonated form of H97 has a higher free energy of 13.9 and 4.1 kcal/mol with and without Mg_2_, respectively.

In the 4 ns MD simulations with a neutral form of H97, the residue H97 stayed in the inactive position and made hydrogen bonds by its N*δ* atom with backbone NH groups of Glu98 and Val99, with or without Mg_2_. With a protonated form of H97, the side chain spontaneously rotated within 100 ps of an MD simulation to a position, which closely resembled the orientation in the activated EF-Tu:Ribosome crystal structure (21). The biprotonated H97 hydrogen bonds the catalytic water molecule by a proton on N*δ*. The relevant distances observed in the MD simulations are given in Supplementary data.

Overall, the most stable state is with a neutral form of H97, and GTP as a mixture of protonated and deprotonated forms with Mg_1_ only. The presence of the Mg_2_ changes the preference for the state with a deprotonated GTP.

### QM/MM free energy simulations of the catalytic reaction

To perform calculations, H97 was first rotated in its activated position as seen in the EF-Tu:ribosome complex (21) (Figure 3a). According to the results using protonation free energy simulations of H97 and GTP, the first mechanism studied involved a neutral H97 acting as a base and a deprotonated GTP (Figure 3a). This mechanism corresponds to that previously proposed for the EF-Tu:Ribosome complex, based on the crystal structure (21). In mechanism I, H97 takes a proton from the water molecule, and the resulting hydroxide attacks GTP, to give a product state consisting of H97^+^ and Pi^2−^. In the presence of Mg_1_ only, the reaction is barrierless with the energy of the product of 34.3 kcal/mol. In the presence of Mg_1_ and Mg_2_ the reaction barrier is 15.7 kcal/mol with the product being more stable than the transition state by 2.7 kcal/mol (Table 4). This value is lower than the experimental estimate for the energy barrier of the complete reaction 20.5 kcal/mol (calculated from rate constant in Table 1). However, this discrepancy may be accounted for by the manual rotation of the side chain of H97 in the simulations. Thus, Mg_2_ strongly stabilizes the transition state and product state by 18.6 kcal/mol and 21.3 kcal/mol, respectively. This stabilization can be understood by interactions between the positive magnesium ion and Pi^2 −^ forming during this reaction. The distances between a water proton and N*δ* of H97 are 1.8, 1.4 and 1.1 Å in the reactant, transition state and product, respectively. The distances between the water oxygen and γ phosphate are 3.0, 2.0 and 1.8 Å in the reactant, transition state and product, respectively. Thus, the proton transfer is concerted with nucleophilic attack of the catalytic water molecule on the γ phosphate of GTP (Figure 3a).

**Table 4.** Energies of intermediate and transition state structures from the proposed mechanisms of GTP activation

Mechanism	H97	Structure	Mg ions	
	protonation		Mg_1_	Mg_1_+Mg_2_
I	His*ϵ*	TS	34.3	15.7
		Product	34.3	13.0
II	His(+)	TS	54.9	50.2
		Product	17.2	14.1
III	His*δ*	TS	38.4	48.2
		Product	1.2	8.2
IV	His*ϵ*	TS	41.1	44.0
		Product	-8.1	-6.9
V	His(+)	TS1	2.9	7.7
		IS	-0.1	7.0
		TS2	26.5	36.6
		Product	1.1	9.0
VI	His*ϵ*	TS	34.6	32.7
		Product	3.1	11.5

All energies are in kcal/mol and are relative to the reactant species. Mechanisms are described in the text and illustrated in Figure 2.

Mechanism II was proposed by (58) for the EF-Tu:ribosome complex, based on simulations with an empirical valence bond potential. In this mechanism, the catalytic histidine would contribute to an allosteric effect rather than act as a general base (Figure 3b). It involves transfer of a proton from the hydrolytic water to the phosphate, followed by hydroxide attack on the terminal phosphate of GTP, to give GDP and Pi^−^ (Figure 3b). With Mg_1_ only and protonated H97, the computed energy barrier for this reaction was 54.9 kcal/mol. No stable intermediate was found. The high energy barrier for the reaction is explained by the need to bring two negative species together. In the presence of Mg_2_ the barrier is 50.2 kcal/mol, which is still too high. Variants of mechanism II but with a neutral histidine (N*δ* or N*ϵ*, Table 4) can also be envisaged (mechanisms III and IV). The reaction paths are very similar with the barrier of 38.4 and 41.1 kcal/mol when H97 is protonated on N*δ* and N*ϵ* respectively. In the presence of Mg_2_ the barrier is 48.2 and 44.0 kcal/mol with H97 protonated on N*δ* and N*ϵ* respectively. Therefore, mechanisms II, III and IV appear to be even more disfavored by the presence of Mg_2_.

Another mechanism studied corresponds to mechanism V in which H97 assists in the reaction. This mechanism was recently proposed for the GTP hydrolysis by the EF-Tu:Ribosome complex based on the QM/MM simulations (24). It starts with a charged form of H97. In the first step, the histidine donates its N*δ* proton to the terminal phosphate of GTP via the hydrolytic water molecule. In the second step of the reaction, the water molecule gives its proton to H97 and attacks the phosphate of GTP in a concerted fashion (Figure 3b). It is interesting to note that this step of the reaction is equivalent to mechanism I, in which H97 acts as a general base, except that the GTP is protonated. The energy barriers for mechanism V are given Table 4. In the presence of Mg_2_ the barrier for second step (26.5 kcal/mol with Mg_1_ only) further increases to 36.6 kcal/mol. Finally, we note that PB calculation shows that H97 may become protonated in the absence of Mg_2_. In this case (mechanism VI) the catalytic mechanism requires a neutral H97 protonated on Nϵ, and exactly corresponds to the second step of mechanism V. Thus, in the absence of Mg_2_ it appears that only this mechanism is energetically possible.

The last mechanism studied corresponds to mechanism VI in which H97 is in the inactive position and involves a second water molecule. In this reaction in contrast to mechanisms II–IV, a proton is shifted through a second water molecule. The computed activation energy is 32.7 and 34.6 kcal/mol with and without Mg_2_. This energy is very high in comparison with the energy of mechanism I in the presence of Mg_2_ and mechanism V in the absence of Mg_2_.

Overall, the results obtained using MD simulations indicate that, at least outside from the ribosome, the presence of Mg_2_ renders a GTP hydrolysis pathway corresponding to mechanism I plausible. In this mechanism, the carboxyl group of D19 residue stabilizes Mg_2_ by strong electrostatic interactions. Without these, the ion would be unstable at this position, as it is shown by its absence in the crystal structure of the D19A mutant (Figure 2c). Therefore to study the influence of D19 in the stabilization of the transition state, a mechanism with a single magnesium ion, which directly interacts with GTP, was considered. The mechanism is equivalent to mechanism V of the wild-type protein, for which the computed barrier is 26.5 kcal/mol. For D19A we computed the barrier of mechanism V to be higher: 28.1 kcal/mol. The other mechanisms, i.e. proposed by (58) II, III and IV, were not considered due to the very high energy barriers found for the wild-type protein. Mechanism VI involving a second water molecule has a higher activation energy of 34.6 kcal/mol, and thus, less likely than mechanism V in the D19A mutant.

In summary, D19 can accelerate the reaction by changing the protonation state of the GTP (accelerate proton binding) in mechanism V in the absence of Mg_2_ or/and it can accelerate the reaction by providing stabilization of Mg^2+^ ion (accelerate magnesium ion binding) in mechanism I in the presence of Mg_2_.

Finally, the residual GTPase activity observed with H97A (Table 1 and H97AGTP_24_ structure) suggested that aIF2γ provides an overall GTP environment favorable to hydrolysis. The slow reaction in H97A may involve access to the active site of additional water molecules facilitated by the mutation.

## DISCUSSION

The present study shows that H97 and D19 both participate in GTP hydrolysis by aIF2. Determinations of high-resolution crystal structures of aIF2γ variants obtained in the presence of GTP reveal the presence of a second magnesium binding site involving the D19 residue from the GKT loop, strictly conserved in archaeal aIF2. The magnesium ion was not observed when GDPNP was used during crystallization instead of GTP. This reinforces the idea that GDPNP is not a perfect mimic of GTP (54,55) and may explain why Mg_2_ was never observed in previously determined crystal structures. In order to explore a possible role of this magnesium ion in GTP hydrolysis, we used molecular simulation techniques. To perform our simulations, we assumed that GTP hydrolysis in aIF2 involves a motion of the catalytic H97 residue in an activated position as observed for the corresponding H85 residue in the EF-Tu:ribosomal complex (21; Figure 3a). From these calculations, it appeared that in the presence of Mg_2_ the catalytic histidine prefers to be in a neutral form and, thus, is pre-organized to abstract a proton from the catalytic water molecule. In this mechanism, where H97 acts as a catalytic base, the second magnesium would strongly stabilize the negative species forming in the transition and product states. The role of D19 would be associated with its ability to stabilize Mg_2_ bound to the phosphate group of GTP through water-mediated interactions.

**Figure 4.**
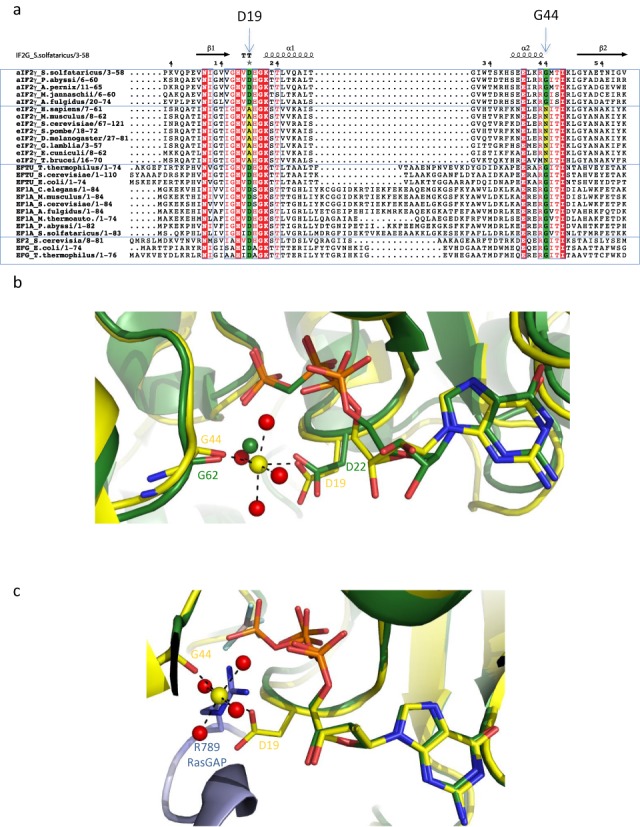
Conservation of D19. (**a**) Multiple sequence alignment of eukaryotic and archaeal e/aIF2γ and of elongation factors in the GKT loop and SW1 regions. The secondary structure elements of Ss-aIF2γ are schematized at the top. Each group of factors is boxed; from top to bottom: archaeal aIF2γ, eukaryotic eIF2γ, elongation factors 1A and elongation factors 2. Residues strictly conserved in all proteins are in red boxes. Residues conserved in most sequences are shown with red letters. D19 and G44 are strictly conserved in archaeal aIF2γ and elongation factors (green boxes). They are replaced by A and N, respectively, in eukaryotic eIF2γ (yellow boxes). The view was drawn with Espript (59). (**b**) Superimposition of H97A-GTP_4_ to EF-G-ribosome complex in a pre-translocation state. The G-domains of EF-G (PDB code 4JUW) and of H97A-GTP_4_ were superimposed. H97A-GTP_4_ is colored in yellow, GTP is colored by atoms with carbons in yellow. The side chain of D19 and the main chain atoms of G44 are shown in sticks. The second magnesium ion is shown as a yellow sphere and the coordinated waters are as red spheres. EF-G is shown in green cartoons. The water molecule liganded by G62 and D22 is shown in green sphere. (**c**) Surperimposition of H97A-GTP_4_ to Ras-RasGAP complex (PDB ID Code 1WQ1, (60)).The G-domains of the two structures were superimposed. H97A-GTP_4_ is colored as in view b. The Ras protein is shown in green cartoons, GDP-AlF_3_ is colored by atoms. RasGAP is shown as a light blue cartoon. The side chain of the catalytic arginine R789 is shown in sticks. The view shows that the guanidinium group of R789 occupies a similar position as that of Mg_2_.

G44 (SW1) and D19 (GKT loop) are strictly conserved in archaeal aIF2γ. However, these two residues are systematically replaced by N and A, respectively, in eukaryotic eIF2γ (Figure 4a, 59). On the other hand, it should be reminded that eukaryotic eIF2 uses a GAP (eIF5) to activate GTP hydrolysis whereas this process is thought to be non-GAP assisted in archaeal aIF2. eIF5 likely acts through stabilization of the transition state by supplying a positively charged arginine residue (R15 in yeast eIF5) to neutralize the negative charges of the phosphate groups (2,14–15). Therefore, the activation mechanism by the GAP would be reminiscent of that described in the Ras-RasGAP complex (60). We superimposed the G-domains of H97AGTP_4_ to that of Ras-RasGAP complex (60). Strikingly, the position of the catalytic arginine residue of RasGAP (R789) matches that of the second magnesium ion bound to D19 (Figure 4c). This observation, as well as the results obtained using molecular simulation techniques, leads us to propose that, in archaea, the second magnesium ion bound to D19 could play a similar role to the one played *in trans* by the catalytic arginine residue of the GAP.

Notably, an aspartate residue is almost systematically found at this position in elongation factors EF1A and EF2 (Figure 4a). Recently, the structure of EF-G bound to the ribosome in an intermediate state of translation was determined (32,33). The equivalent aspartate has a conformation highly similar to that found in aIF2γ, where it coordinates a water molecule close to the γ-phosphate of GDPCP (Figure 4b). Moreover, just after completion of the present manuscript, an article by Kuhle and Ficner was made available on line (61). Starting from a high-resolution structure of eIF5B bound to GTPγS, the authors provide evidence that eIF5B and aEF1A/EF-Tu coordinate a monovalent cation in the same area as the one described here for Mg_2_. Coordination involves the conserved aspartate, although it differs from that for Mg_2_. Indeed, the monovalent cation is directly bound to three phosphoryl oxygens whereas Mg_2_ is linked to phosphoryl oxygens through a bridging water molecule (Figure 3a). Conclusions from (61), from another study appeared during revision of our manuscript (62) and from the present study are fully in line. Together, these studies strongly indicate that non-GAP assisted translational GTPases use previously unidentified positive charges for GTP hydrolysis, brought either by a monovalent cation for GTPases acting on the assembled ribosome or by a second magnesium ion for aIF2, a factor that acts on the 30S ribosome.

This study highlights the important role played by the aspartate residue of the GKT loop. In archaeal aIF2, this residue bound to Mg_2_ may explain how GTP hydrolysis may occur without GAP assistance. In the present work, GTP hydrolysis by aIF2 was studied outside from the ribosome. Up to now, we failed to observe a significant acceleration of GTP hydrolysis in *in vitro* reconstituted translation initiation assays using archaeal components. Therefore, the mechanism by which GTP hydrolysis is activated, likely through transition of H97 from its rest position to its activated position remains to be elucidated. In particular, contrarily to other translational GTPases, e/aIF2 GTPase activity is not modulated through interaction with the GTPase center on the assembled ribosome but within a pre-initiation complex on the small ribosomal subunit. Hence, it remains possible that during translation initiation, interaction of aIF2γ with other partners occurs. In EF-Tu, H85 is rotated in its activated position by interaction with the phosphate of residue A2262 of the sarcin-ricin loop (21). The negative charge carried by the phosphate group of A2262 favors a biprotonated state for H85. In the case of aIF2, we show that Mg_2_ allows the occurrence of a GTP hydrolysis mechanism where H97 is neutral. This makes it possible that H97 is activated by a fundamentally different mechanism, not involving ribosomal RNA. Clearly, further studies are needed to understand how this process is regulated on the ribosome and how the release of the Pi group can be coupled with AUG recognition.

This work also brings some clues about the mechanism by which aIF2 is released from the tRNA after Pi release. Now, several Ss-aIF2 structures are available in different nucleotide states and, importantly, in different space groups. As already noticed (27,50), transition from switch ON to switch OFF is systematically accompanied by rotation of domains II and III with respect to domain I. This is a strong indication that the rotation is indeed linked to the switch motions and not to crystal packing constraints. Therefore, on the ribosome, Pi release would trigger the OFF state of aIF2, thereby disrupting the tRNA binding site through rotation of domains II and III. In this process R219 would play an important role both by positioning domain II relative to domain I and by interacting with the terminal adenosine of the tRNA. In agreement with this idea, mutation of the corresponding R in yeast eIF2γ (R319) caused loss of eIF2 function (4).

## ACCESSION NUMBERS

The Protein Data Bank accession numbers for the structures reported in this paper are 4RCY, 4RCZ, 4RD0, 4RD1, 4RD2, 4RD3, 4RD4 and 4RD6.

## SUPPLEMENTARY DATA

Supplementary Data are available at NAR Online.

SUPPLEMENTARY DATA
