# Supplementary material for: Identification of a second GTP-bound magnesium ion in archaeal initiation factor 2
Source: Nucleic Acids Res. 2015 Feb 17;43(5):2946–57. doi: 10.1093/nar/gkv053 (PMC4357699; doi:10.1093/nar/gkv053)
Supplement: SUPPLEMENTARY DATA [file supp_43_5_2946__index.html]

Identification of a second GTP-bound magnesium ion in archaeal initiation factor 2 — Identification of a second GTP-bound magnesium ion in archaeal initiation factor 2 — SUPPLEMENTARY DATA 

# Identification of a second GTP-bound magnesium ion in archaeal initiation factor 2

## SUPPLEMENTARY DATA

**Files in this Data Supplement:**

- SUPPLEMENTARY DATA
