# Supplementary material for: Identification of a second GTP-bound magnesium ion in archaeal initiation factor 2
Source: Nucleic Acids Res. 2015 Feb 17;43(5):2946–57. doi: 10.1093/nar/gkv053 (PMC4357699; doi:10.1093/nar/gkv053)
Supplement: SUPPLEMENTARY DATA [file supp_gkv053_Supplemental_information-revision-formatted.docx]

Identification of a second GTP-bound magnesium ion in

archaeal initiation factor 2

Etienne Dubiez, Alexey Aleksandrov, Christine Lazennec-Schurdevin, Yves Mechulam and Emmanuelle Schmitt

Laboratoire de Biochimie, Ecole Polytechnique, CNRS, F-91128 Palaiseau cedex, France

**Supplementary data**

**Supplementary Material and Methods**

*Poisson-Boltzmann Linear Response Approximation (PBLRA)*

For the protonation free energy of the GTP gamma phosphate and H97, in the aIF2 complex and alone in solution, we used a simpler Poisson-Boltzmann Linear Response Approximation, or PB/LRA ([1](#_ENREF_1)). Protonations of the phosphate group and imidazole ring of H97 were modeled by changing selected atomic charges. The corresponding free energy change was computed both in the protein complex and for the ligand alone in solution, using conformations taken from a 2 ns aIF2γ:GTP MD simulations. The free energy was approximated by the continuum electrostatic free energy, where the protein and ligand atoms are explicitly included but the solvent is replaced by a dielectric continuum. The free energy change in either medium (protein, solution) can be written as:

ΔG = ∑*δq_i_ (<V_i_>_A_+<V_i_>_B_) / 2*

The sum is over all the atoms of the titrable group; *δq_i_* is the change in the atomic charge due to the protonation; *V_i_* is the electrostatic potential on atom *i* when the proton charge is absent. The brackets represent averaging over the ensemble of conformations drawn from an MD simulation when the phosphate is protonated or deprotonated (one snapshot every 20 ps over the last 4 ns of MD). The electrostatic potentials were computed for each MD conformation by numerically solving the Poisson-Boltzmann equation of continuum electrostatics, where the protein and ligand were treated as a single dielectric medium with a dielectric constant of 2; solvent was treated as another medium with a dielectric constant of 80, the experimental value for bulk water. A low dielectric value is appropriate for the protein because the conformational changes induced by the proton binding are explicitly modeled by simulating the protonated/deprotonated states ([1](#_ENREF_1),[2](#_ENREF_2)).

The boundary between the two dielectric media was defined as the protein/ligand molecular surface, computed with a 1.6 Å radius probe sphere. For the potential calculation, the system was discretized using a cubic grid with a 68 Å edge and a spacing of 0.4 Å. The linearized Poisson-Boltzmann equation was solved with Coulombic boundary conditions, using the Charmm program ([3](#_ENREF_3)). We used a ionic strength corresponding to a 0.15 M concentration of monovalent ions. Two waters coordinating the Mg_1_ ion, and four waters coordinating the Mg_2_ ion, and catalytic water molecule were treated explicitly. The same procedure was followed for GTP in water, using the conformations drawn from the protein simulations. Two waters that coordinate the Mg^2+^ were treated explicitly, as part of the ligand. The pKa shift due to the protein environment has the form:

pKa,prot – pKa,solv = (ΔGprot – ΔGsolv) / 2.303 *kT*

*QM/MM reaction path free-energy perturbation calculations*

We estimated free energies of the structures along the NEB pathways using the free energy perturbation (FEP) method of Kaestner et al ([4](#_ENREF_4)), which itself is similar to that of Zhang and co-workers ([5](#_ENREF_5)). The system setup was similar to that described in section Molecular Dynamics Simulations, except that the atoms in the QM region and the link atoms were fixed at their corresponding optimized geometries from the NEB profile. CHELP ([6](#_ENREF_6)) charges, computed with the B3LYP/def2-TZVP DFT method, were used for the atoms in the QM region and CHARMM27 charges for those in the MM region. This approximation was shown to give a good result in comparison to the results obtained with the full self-consistent field iterations in each MD step ([4](#_ENREF_4)).

For each image of the NEB profiles a MD simulation was performed at constant room temperature and pressure for 500 ps. This gave 10 ns of simulation total. The free-energy change between image *i* and *i*+1 due to the QM/MM interactions was calculated according to the formula:

Δ*A*^i→i+1^= – - 1/ *β* ln<exp(-*β* ∆E_pert_^i→i+1^)>_mm,i_

where E_pert_^i→i+1^ is the energy of perturbation computed with the MM atoms coordinates from the MD simulation of image *i*, and the QM positions of image *i*+1. Following the previous work ([4](#_ENREF_4),[5](#_ENREF_5)), estimates of the free energies of the atoms in the QM region were obtained using a rigid-rotor harmonic-oscillator approximation after normal mode analysis. However, we found that these contributed negligibly to the calculated free energy differences between the different structures.

**Supplementary Table**

|  | MD simulations | | | | X-Ray |
| --- | --- | --- | --- | --- | --- |
|  | Mg_1_ | | Mg_1_+Mg_2_ | |  |
|  | H97*ε* | H97(+) | H97*ε* | H97(+) |  |
| P*γ* (GTP)… O (Wat) | 3.4 (0.1) | 3.4 (0.1) | 3.5 (0.1) | 3.5 (0.1) | 3.7 |
| N*δ* (H97) … O (Wat) | 6.2 (0.3) | 2.8 (0.1) | 6.0 (0.3) | 2.8 (0.1) | 6.0 |
| N*δ* (H97) … N (E98) | 3.1 (0.2) | 5.5 (0.1) | 3.2 (0.2) | 5.4 (0.1) | 3.0 |
| N*δ* (H97) … N (V99) | 3.5 (0.4) | 7.1 (0.2) | 3.8 (0.5) | 6.9 (0.2) | 3.1 |

**Table S1:** Selected distances between atoms in MD model and X-ray structure. Distance fluctuations are in parentheses.

**Supplementary Figures**


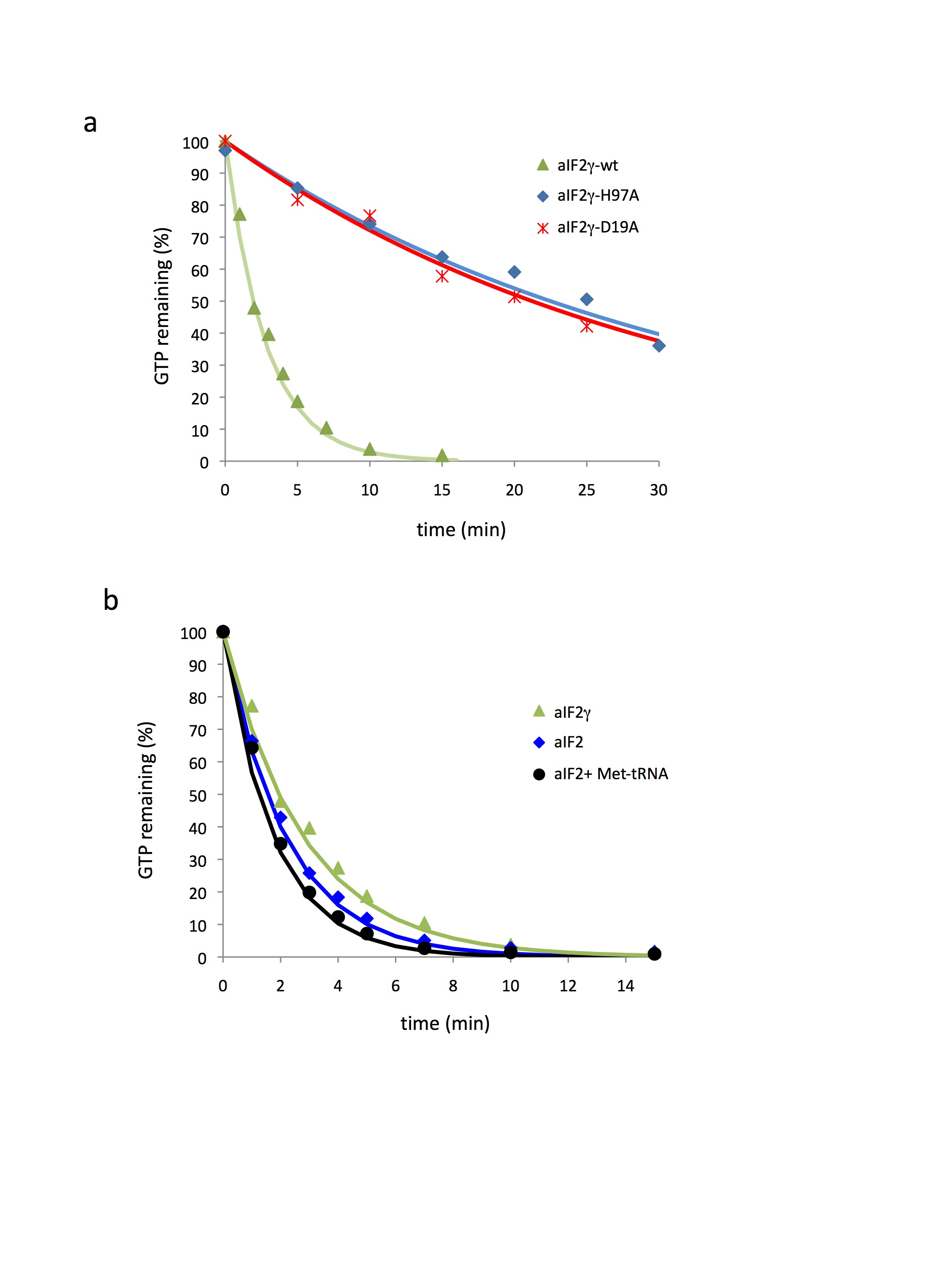


**Figure S1:** Plots of GTP hydrolysis kinetics for aIF2 and aIF2 variants. Experiments were performed as described in Material and Methods. For each studied protein, the results from a representative experiment are shown. Each experiment was repeated at least three times for rate determination (Table 1). The experimental points are shown together with the corresponding fitted exponential curve. Values are expressed as percentage of GTP remaining relative to total γ[^32^P]-GTP initially added in the assay. Plotted zero time points actually correspond to a few seconds of incubation.

1. Effects of H97A and D19A mutations on the rates of GTP hydrolysis by the aIF2γ subunit.
2. Comparison of GTP hydrolysis for complete heterotrimeric aIF2 (aIF2), the isolated γ subunit (aIF2γ) and aIF2 in the presence of Met-tRNA_f_^Met^A1-U72 (aIF2+Met-tRNA).

**
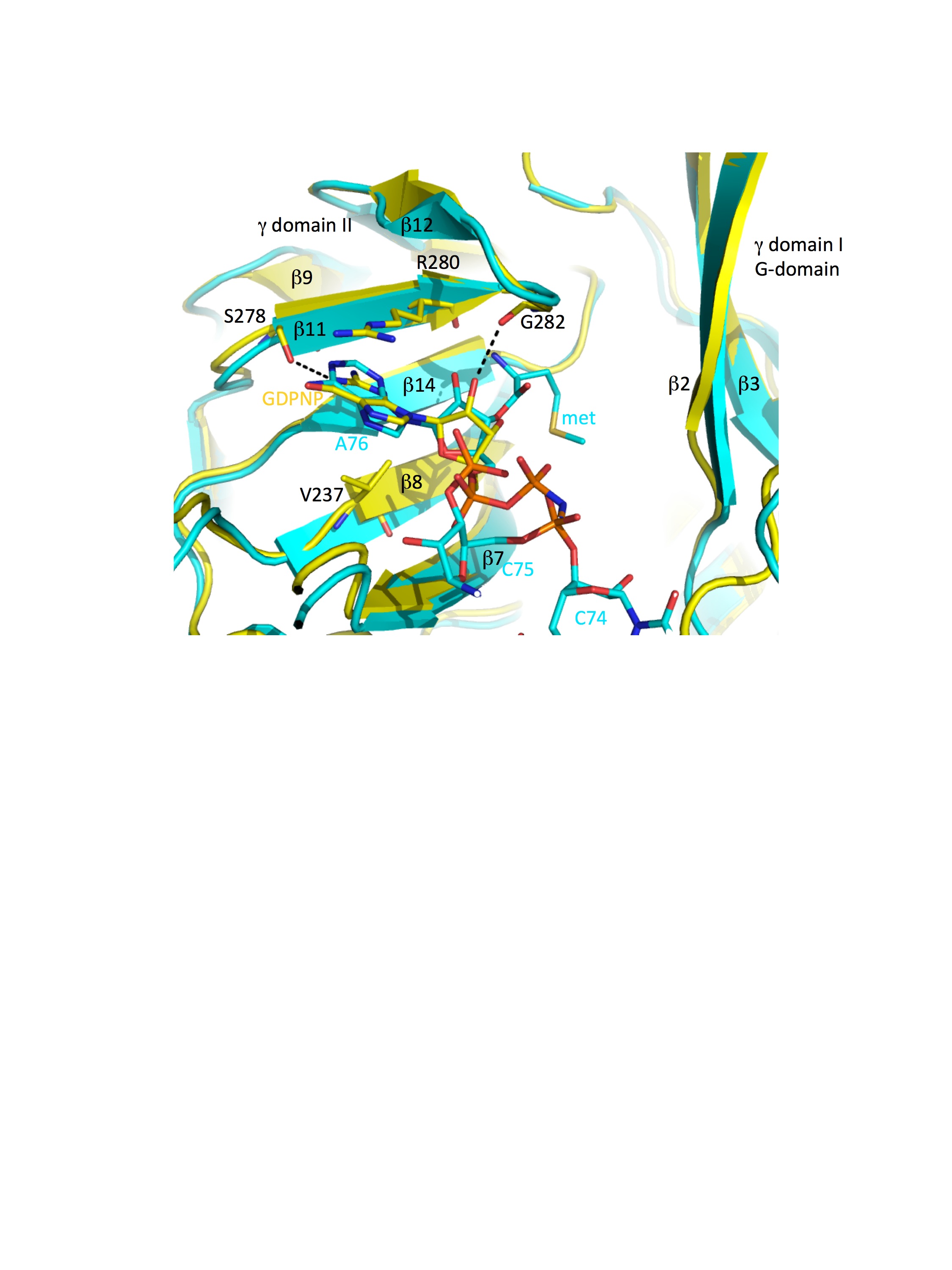
**

**Figure S2:** Secondary GDPNP binding site.

WT-aIF2γ-GDPNP structure was superimposed on the structure of the ternary aIF2-GDPNP-Met-tRNA complex (PDB ID Code 3V11, ([7](#_ENREF_7))). WT- aIF2γ-GDPNP structure is yellow and the γ subunit from 3V11 is cyan. The tRNA is drawn in cyan sticks. The view shows that the second GDPNP binding site corresponds to the binding site of the terminal A76 base of the met-tRNA bound to aIF2. S278, R280, G282, V237 are involved in the binding of the pyrimidin rings in both structures. For the sake of clarity, only the residues of wt-aIF2γ-GDPNP are shown (yellow sticks). According to the refined B values, this secondary guanine nucleotide binding site is likely to be a weaker affinity site as compared to the canonical site (Table 2).


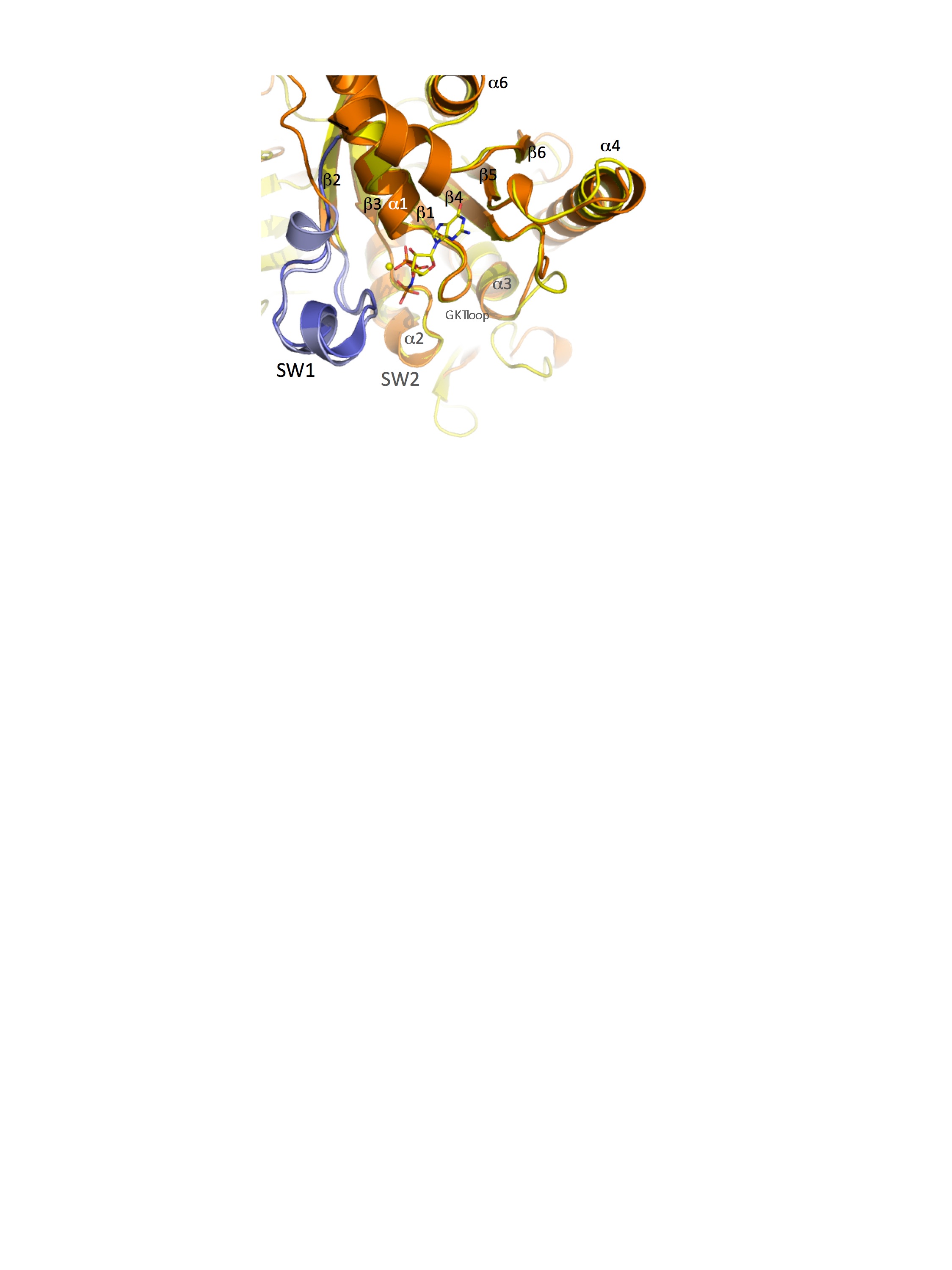


**Figure S3:** Comparison of the structure of aIF2γ with that of EF-Tu.

Domain I of wt-aIF2γ-GDPNP structure (residues 1-210) was superimposed on the G domain of *T. thermophilus* EF-Tu bound to GDPNP and tRNA (PDB ID Code 1B23, ([8](#_ENREF_8))) with an rmsd value of 1.07 Å for 117 pairs of Cα atoms compared. Wt-aIF2γ-GDPNP is yellow and EF-Tu is orange. Switch 1 region of wt-aIF2γ-GDPNP is slate blue whereas switch 1 region of EF-Tu is light blue. Secondary structure elements are labeled according to wt-aIF2γ-GDPNP ([9](#_ENREF_9)). For the sake of clarity, only the GDPNP molecule bound to aIF2γ was drawn in sticks. The view highlights the high similarity of the SW regions in EF-Tu and aIF2γ in their GDPNP-bound forms.


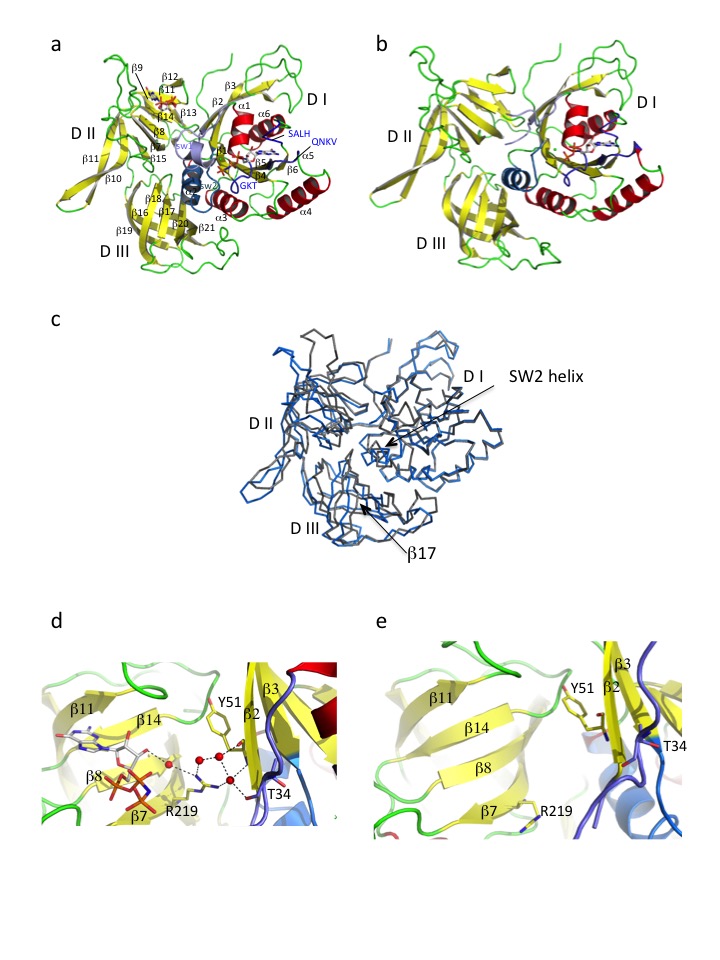


**Figure S4:** Comparison of wt-aIF2γ-GDPNP with wt-aIF2γ-GDP

a-wt-aIF2γ-GDPNP

b-wt-aIF2γ-GDP

In both view structures are shown in ribbon and colored according to their secondary structure elements. Liganded nucleotides are shown in sticks. Secondary structure elements are labeled as in ([9](#_ENREF_9)). Sw1, sw2, GKT, QNKV, SALH regions are colored in blue and labeled.

c-ribbon representation of wt-aIF2γ-GDPNP superimposed on wt-aIF2γ-GDP. Domains I (DI) of the two structures were superimposed with an rms value of 0.665 Å for 1134 atoms compared. Wt-aIF2γ-GDPNP is in blue and wt-aIF2g-GDP is in grey.

d-Close-up view of the secondary nucleotide binding site of wt-aIF2γ-GDPNP. The view shows tight interactions involving R219 of domain II and water molecules with T34 and Y51 from the switch 1 region of domain I. Notably, a same position of R219 is observed in 4M53 ([10](#_ENREF_10)) or in 2AHO ([9](#_ENREF_9)). Since 2AHO does not contain a nucleotide at the secondary site, the position of R219 is linked to the switch ON state rather than to occupation of the secondary site.

e-Same view as d. but for wt-aIF2γ-GDP. R219 adopts a relaxed position and does not contact domain I.
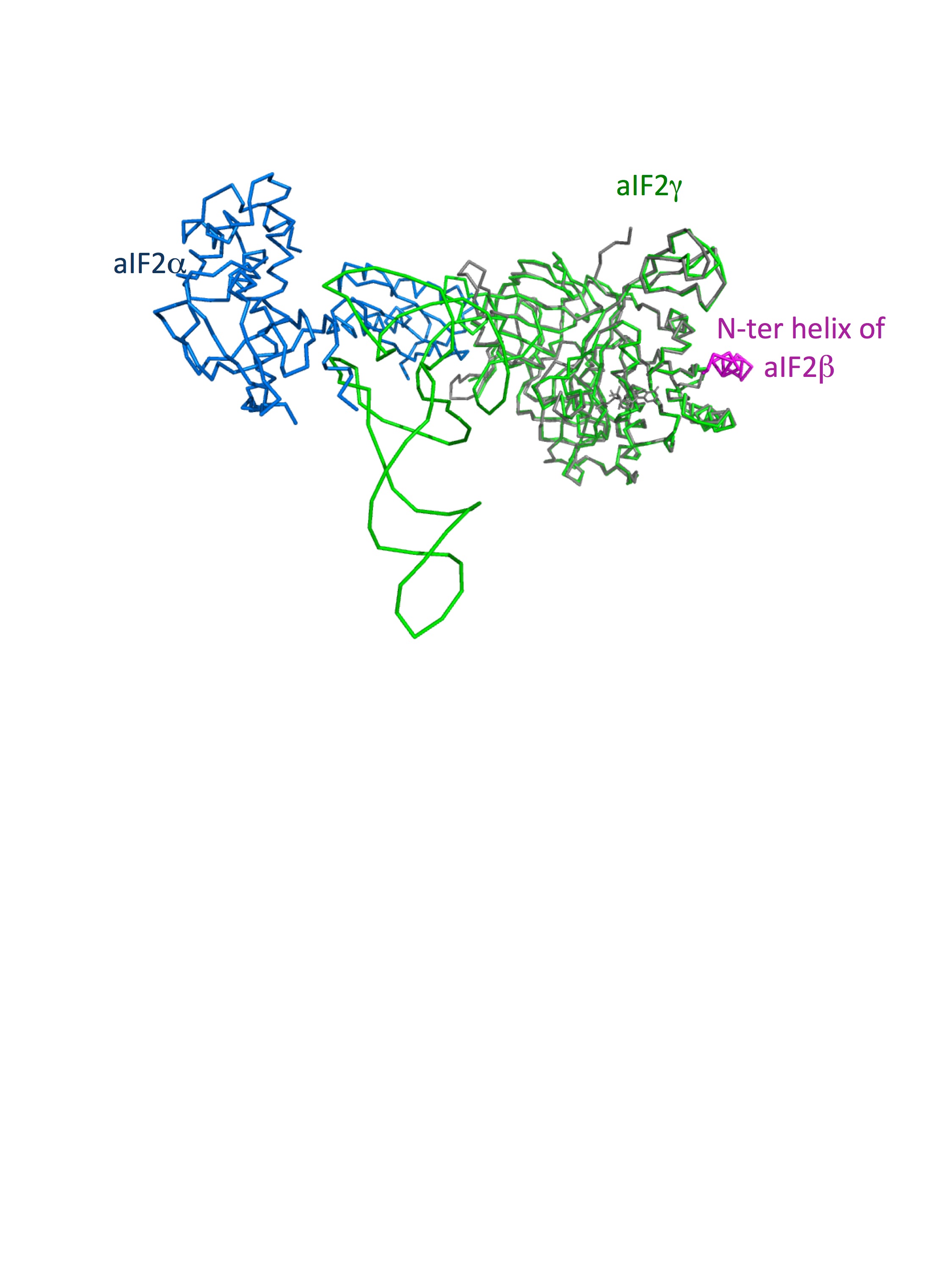


**Figure S5:** Comparison of wt-aIF2γ-GDPNP with aIF2 bound to GDPNP and tRNA (PDB ID code 3V11, ([7](#_ENREF_7))).

Ribbon representation of wt-aIF2γ-GDPNP (gray) superimposed on aIF2γ from 3V11. 3V11 is colored as follows: γ in green, α in blue, the N-terminal helix of beta in magenta and the tRNA in green. The two aIF2γ subunits are superimposed with an rms value of 0.595 for 358 Cα atoms compared.


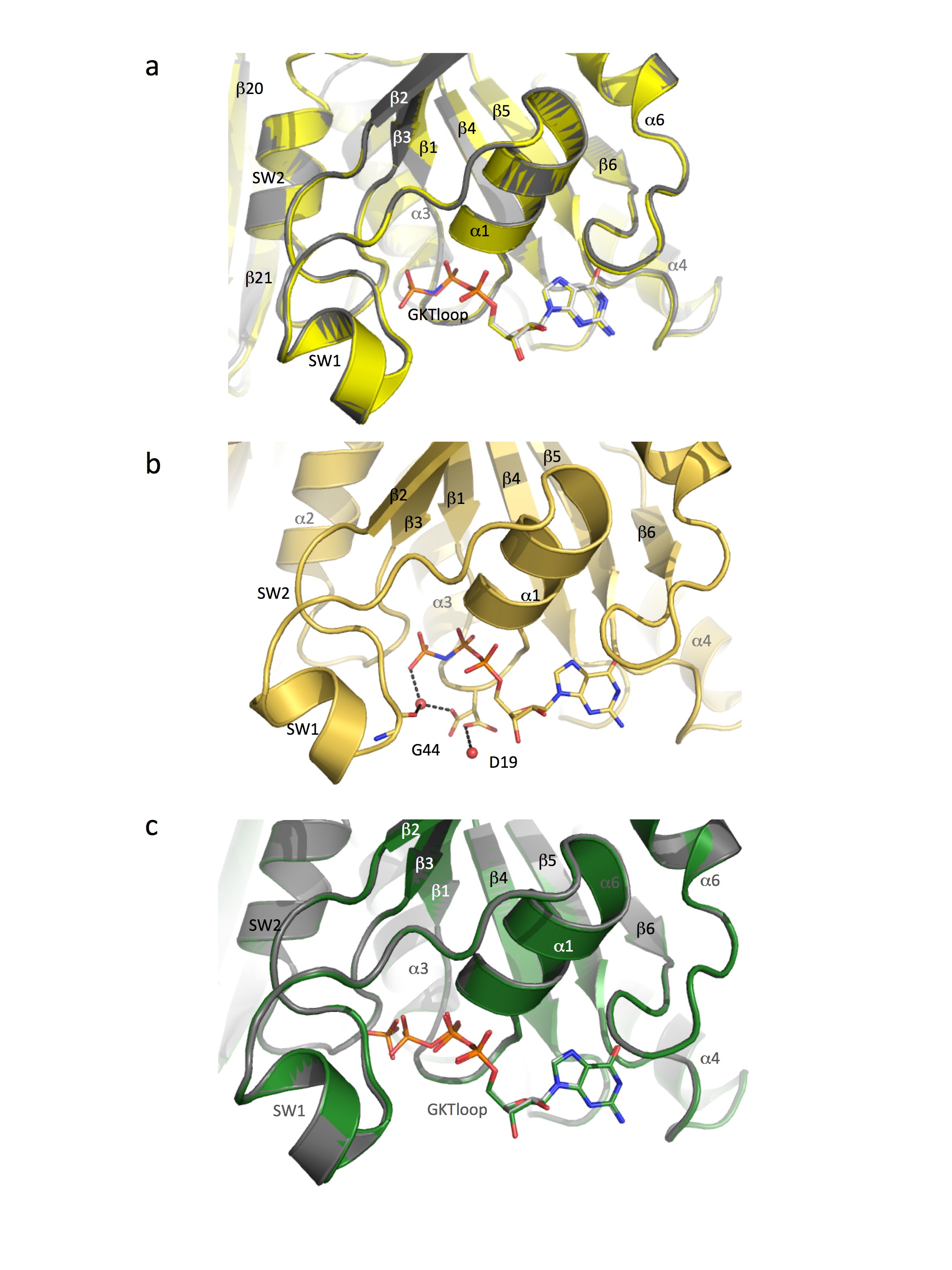


**Figure S6:** Comparison of WT and HA97 aIF2γ structures.

a- Comparison of wt-aIF2γ-GDPNP with H97A-GTP_4_. The G domains were superimposed with an rmsd value of 0.15 Å for 1311 atoms compared. WT-aIF2γ−GDPNP is yellow and H97A-GTP_4_ is grey. GDPNP and GTP are shown in sticks (carbon atoms in yellow and grey, respectively)

b- Close-up view of H97A-GDPNP structure. H97AGDPNP is shown in yelloworange cartoons. Alternative side chain positions of D19 and main chain atoms of G44 are shown in sticks. Water mediated interactions involving the two residues are shown.

c: Comparison of H97AGTP_4_ with H97AGTP_24_.

The G domains were superimposed with an rmsd value of 0.096 Å for 1285 atoms compared. H97AGTP_4_. is grey and H97AGTP is green. GTP and GDP:Pi are shown in sticks (carbon atoms in grey and green, respectively)

References

1. Aleksandrov, A., Proft, J., Hinrichs, W. and Simonson, T. (2007) Protonation patterns in tetracycline:tet repressor recognition: simulations and experiments. *Chembiochem*, **8**, 675-685.

2. Sham, Y., Chu, Z. and Warshel, A. (1997) Consistent calculations of pKa's of ionizable residues in proteins: semi-microscopic and microscopic approaches. *J. Phys. Chem.*, **B101**, 4458-4472.

3. Im, W., Beglov, D. and Roux, B. (1998) Continuum solvation model: computation of electrostatic forces from numerical solutions to the Poisson-Boltzmann equation. *Comp. Phys. Comm.* , **111**, 59-75.

4. Kästner, J., Senn, H.M., Thiel, S., Otte, N. and Thiel, W. (2006) QM/MM Free-Energy Perturbation Compared to Thermodynamic Integration and Umbrella Sampling: Application to an Enzymatic Reaction. *J. Chem. Theory Comput.*, **2**, 452-461.

5. Zhang, Y., Liu, H. and Yang, W. (2000) Free energy calculation on enzyme reactions with an efficient iterative procedure to determine minimum energy paths on a combined ab initio QM/MM potential energy surface. *J. Chem. Phys.*, **112**, 3483-3492.

6. Breneman, C.M. and Wiberg, K.B. (1990) Determining atom-centered monopoles from molecular electrostatic potentials. The need for high sampling density in formamide conformational analysis. *J. Comput. Chem*, **11**, 361-373.

7. Schmitt, E., Panvert, M., Lazennec-Schurdevin, C., Coureux, P.D., Perez, J., Thompson, A. and Mechulam, Y. (2012) Structure of the ternary initiation complex aIF2-GDPNP-methionylated initiator tRNA. *Nat. Struct. Mol. Biol.*, **19**, 450-454.

8. Nissen, P., Thirup, S., Kjeldgaard, M. and Nyborg, J. (1999) The crystal structure of Cys-tRNACys-EF-Tu-GDPNP reveals general and specific features in the ternary complex and in tRNA. *Structure*, **7**, 143-156.

9. Yatime, L., Mechulam, Y., Blanquet, S. and Schmitt, E. (2006) Structural switch of the gamma subunit in an archaeal aIF2 alpha gamma heterodimer. *Structure*, **14**, 119-128.

10. Nikonov, O., Stolboushkina, E., Arkhipova, V., Kravchenko, O., Nikonov, S. and Garber, M. (2014) Conformational transitions in the gamma subunit of the archaeal translation initiation factor 2. *Acta Crystallogr D Biol Crystallogr*, **70**, 658-667.
